# Study of CTS DNA Proficiency Tests with Regard to DNA Mixture Interpretation: A NIST Scientific Foundation Review

**DOI:** 10.3390/genes13112171

**Published:** 2022-11-21

**Authors:** Todd Bille, Michael D. Coble, Tim Kalafut, John Buckleton

**Affiliations:** 1 National Laboratory Center, 6000 Ammendale Road, Beltsville, MD 20705, USA; 2 Center for Human Identification, Department of Microbiology, Immunology, and Genetics, University of North Texas Health Science Center, 3500 Camp Bowie Blvd., Fort Worth, TX 76107, USA; 3 Department of Forensic Science, College of Criminal Justice, Sam Houston State University, Huntsville, TX 77340, USA; 4 Institute of Environmental Science and Research Limited, Private Bag 92021, Auckland 1142, New Zealand; 5 Department of Statistics, University of Auckland, Private Bag 92019, Auckland 1142, New Zealand

**Keywords:** Forensic DNA interpretation, proficiency testing, probabilistic genotyping, CTS

## Abstract

The National Institute of Standards and Technology has released a document entitled DNA Mixture Interpretation: A NIST Scientific Foundation Review for public comment. This has become known as the Draft NIST Foundation Review. It contains the statement: “Across these 69 data sets, there were 80 false negatives and 18 false positives reported from 110,408 possible responses (27,602 participants × two evidence items × two reference items). In the past five years, the number of participants using PGS has grown.” We examine a set of proficiency test results to determine if these NIST statements could be justified. The summary reports for each relevant forensic biology test (Forensic Biology, Semen, and Mixture) in the years 2018–2021 were reviewed. Data were also provided to us by CTS upon our request. None of the false positives or negatives could be attributed to the mixture interpretation strategy and certainly not to the use of PGS.

## 1. Introduction

In June of 2021, a group of authors from The National Institute of Standards and Technology released a document entitled DNA Mixture Interpretation: *A NIST Scientific Foundation Review* for public comment [1]. This has become known as the Draft NIST Foundation Review. 

The Draft NIST Foundation Review [1] contains a section on the NIST review team’s reporting of proficiency test results (starting on page 75). This includes the statements:

“Across these 69 data sets, there were 80 false negatives and 18 false positives reported from 110,408 possible responses (27,602 participants × two evidence items × two reference items). In the past five years, the number of participants using PGS has grown”.

It is possible to infer from the above statement that Probabilistic Genotyping Software (PGS or PG) contributed to the false positives or negatives. 

The Draft NIST Foundation Review concludes: “KEY TAKEAWAY #4.1: The degree of reliability of a component or a system can be assessed using empirical data (when available) obtained through validation studies, interlaboratory studies, and proficiency tests”.

We examine a set of proficiency test results to determine if both of these NIST statements could be justified.

## 2. Method

Collaborative Testing Services, Inc. (CTS) publish the results of their proficiency tests on the internet at https://cts-forensics.com/program-1.php accessed on 8 October 2022. The CTS forensic biology proficiency tests provide four samples (either as samples or profiles): Items 1 and 2 serve as references for comparison to questioned items 3 and 4. A mock case scenario is also provided.

Respondents are asked to provide the genotyping results of the four samples and a statement such as: could the Victim (Item 1) and/or the Suspect (Item 2) be a contributor to the questioned samples (Item 3 and Item 4)? Answers are given by ticking a box from the options yes, no, inconclusive, and no interpretation. Therefore, there are potentially four comparisons made by the analyst per test. No statistical analysis is requested.

The manufacturer of the tests provides the consensus of the pre distribution laboratories and at least 10 participating laboratories. These are assumed to be the expected answers.

The summary reports for each relevant forensic biology test (Forensic Biology, Semen, and Mixture) in the years 2018–2021 were searched and those participants recording an answer different from the consensus result were noted. Based on the text of the NIST Draft Foundation, we infer that the NIST team scored a yes where the consensus was “no” as a false positive and vice versa. We follow this procedure but note that this means that the terms false positive and false negative will now include things such as samples where a component was below the detection standard.

Data were provided to us by CTS upon our request. The CTS data analysts were able to mine the data and provide the number of false positive/false negative results per test and the number of reporting PGS labs that fell in the group. For example: “three false negatives, one PGS lab” indicated that two non-PGS labs and one PGS lab reported a false negative for this test. We are limited to what the participating labs actually reported with respect to whether or not they use PGS. Some labs do not use their PGS for the determination of inclusion/exclusion status for a reference. Data were separated based on whether the laboratory was using PGS or not. We point out that this data is imperfect, as there is no requirement for a lab to indicate PGS use on the CTS test. It is entirely at the discretion of the responding laboratories and is based on various internal policies.

We examined the summary report to assign the probable cause of each discordance. Sometimes the participant had given a comment that indicated the reason. In others we were able to see that the genotyping was consistent with the consensus result, but the yes/no was not. We surmise that these were incorrectly filled forms since the inclusion/exclusion in these cases was obvious.

## 3. Results

The results are given in Table 1, Table 2 and Table 3.

Only seven of the discordant results did not have an obvious cause. Five of these appeared to be the result of checking the incorrect boxes for the CTS report. All of the reported profiles for these five instances were consistent with the consensus result. The two remaining instances contained DNA profiles that were not consistent with the consensus results. 

Fifteen discordant results were due to the laboratory only reporting the male fraction/component for a semen-containing stain where the victim was also detected. Therefore, the victim was “excluded” from stains that were known to be a mixture of the victim and the male (semen) component when reporting the results on the CTS form.

There was one instance of possible low-level contamination of an evidence item (Item 3) with a reference item (Item 2), which led to a false inclusion.

In four instances, the female or victim component of a blood/semen mixture was weakly detected in the epithelial fraction. The participants determined that the minor component was not suitable for comparison purposes which resulted in false negative conclusions with respect to these individuals.

The majority of the discordant results were false negatives due to the type of analysis performed. Nineteen participants performed mtDNA analysis on the evidence samples. When these samples contained a mixture of blood and semen, only the mtDNA from the blood component was detected and reported. This resulted in false exclusions of the semen contributor. While these conclusions are not consistent with the consensus results, they are consistent with expected results of mtDNA analysis of this type of mixture.

The final discordant conclusion appeared to be the result of a sample switch. The reported DNA profile of one of the reference samples (Item 2) was a mixture and the reported DNA profile of one of the evidence samples (Item 3) was a single source profile. The consensus result for Item 3 was a mixed DNA profile. 

## 4. Conclusions

The instances of false positives and false negatives that arise from the probable causes: only reported male fraction, minor component (female) of differential epithelial cell fraction not suitable for comparison, and mtDNA from blood/semen mixture are not errors and are not related to PGS in any fashion. The “only reported male fraction” discordant results were concordant with respect to the male fraction. The results of the mtDNA analyses are what one would expect for a blood/semen mixture. The issue with the minor component female is not a problem with interpretation, but instead with the extraction or the original sample set-up. This could sometimes also be affected by where the cutting was taken from the substrate provided in the test. 

Sample switching, contamination, and reporting results incorrectly are serious errors. Part of routine casework is a technical review that would most likely catch these non-PGS related errors. However, none of these have to do with the mixture interpretation strategy and certainly not with PGS.

It is generally considered that the most serious interpretation error in forensic science is that of a false positive, or an erroneous inclusion. According to the data provided by CTS at our request, there were zero false positives among laboratories that used PGS. This information was not available as presented in the Draft NIST Foundation Review. However, we would never claim that PGS use would make a respondent error proof. We merely point this out to remind the reader that the CTS data as presented in the Draft NIST Foundation Review is not suitable for discussion as done by NIST.

In the end, proficiency test data are currently not a good metric to judge the overall reliability of a system. Individual laboratory systems can use the results to determine how the individual participants performed since the labs know the conditions and parameters of their analysis and reporting. In addition, there are no restrictions on who can participate in vendor-provided proficiency tests, meaning these tests can be used for training, research, or academic purposes. Attempting to judge the overall reliability of a discipline/system using proficiency test data without knowing the sources and causes of each discordant result is misleading and uninformative. 

The degree of reliability for PGS, or really any system, cannot be truly assessed by simply examining the numbers that the Draft NIST Foundation Review has presented for proficiency testing results. These numbers can be deceiving and do not truly represent the reliability of a system. If proficiency test data are going to be used to evaluate reliability, a more in-depth examination must be performed.

## Figures and Tables

**Table 1:** An analysis of the CTS forensic biology summary reports for the years 2018–2021 showing the false positive and false negative results for PGS and non-PGS laboratories. Note that the Draft NIST Foundation Review showed only the numbers of false negative and false positive results with no attempt to distinguish PGS/non-PGS responses.

Test Year-Number	Total Participants	Non-PGS Participants	PGS Participants	False Neg Non-PGS	False Pos Non-PGS	False Neg PGS	False Pos PGS
18-5701	740	601	139	1	1	0	0
18-5702	708	540	168	1	0	0	0
18-5703	373	297	76	0	0	0	0
18-5704	722	574	148	1	0	0	0
18-5705	661	468	193	0	0	0	0
18-5706	355	258	97	0	0	0	0
18-5801	156	147	9	3	1	0	0
18-5802	226	180	46	0	0	0	0
18-5803	105	90	15	0	0	0	0
18-5804	181	158	23	1	0	0	0
18-5805	268	221	47	0	0	0	0
18-5806	178	145	33	0	0	0	0
19-5701	732	605	127	0	0	0	0
19-5702	739	480	259	0	0	0	0
19-5703	366	263	103	0	0	0	0
19-5704	726	543	183	1	1	0	0
19-5705	766	485	281	13	1	0	0
19-5706	333	196	137	0	0	0	0
19-5801	168	130	38	0	0	0	0
19-5802	223	177	46	0	0	0	0
19-5803	114	95	19	0	0	0	0
19-5804	166	143	23	2	0	1	0
19-5805	199	185	14	0	0	0	0
19-5806	171	125	46	0	0	0	0
20-5701	671	489	182	0	0	0	0
20-5702	734	427	307	6	0	0	0
20-5703	345	188	157	0	0	0	0
20-5704	728	492	236	1	1	0	0
20-5705	720	406	314	0	0	0	0
20-5706	327	164	163	0	0	0	0
20-5801	235	193	42	1	0	0	0
20-5802	207	172	35	0	0	0	0
20-5803	90	70	20	0	0	0	0
20-5804	186	146	40	0	0	0	0
20-5805	226	192	34	0	0	0	0
20-5806	202	150	52	0	0	0	0
21-5701	635	416	219	0	0	0	0
21-5702	798	459	339	0	0	1	0
21-5703	378	198	180	1	0	0	0
21-5704	590	393	197	3	0	0	0
21-5705	717	515	202	0	0	0	0
21-5706	353	189	164	0	0	0	0
21-5801	167	132	35	0	0	0	0
21-5802	209	180	29	1	1	0	0
21-5803	108	78	30	0	0	0	0
21-5804	210	173	37	5	0	0	0
21-5805	220	180	40	0	0	0	0
21-5806	222	146	76	0	0	0	0
Total	18,654	13,254	5400	41	6	2	0

**Table 2:** The probable cause of the false positive and false negatives given in Table 1. These tally 47 instead of 49, because, in two instances, one occurrence led to both a false positive and a false negative.

Probable Cause	Number of Occurrences
Unknown	2
Reported results incorrectly	5
Only reported male fraction	15
Possible contamination	1
Minor component (female) of differential epithelial cell fraction not suitable for comparison	4
mtDNA from blood/semen mixture	19
Sample switch	1

**Table 3:** Summary statistics for the false positive (FP) and false negatives (FN). The total comparisons were calculated by multiplying the number of participants by the number of comparisons per test (four).

	Total Comparisons	FN Count	FP Count	% FN	% FP
Non-PGS	53,016	41	6	0.077%	0.011%
PGS	21,600	2	0	0.009%	0.000%

## Data Availability

All data analyzed in this study were obtained from Collaborative Testing Services, Inc. (CTS) https://cts-forensics.com/program-1.php (accessed on 28 January 2022 through 24 June 2022).
